# Dosimetric characterization of a Varex VF‐80/μXHP x‐ray unit using open‐ended applicators for superficial radiotherapy

**DOI:** 10.1002/acm2.70326

**Published:** 2025-11-23

**Authors:** Muhammad Zeeshan, Yikai Wu, Zhongyu Qi, Ning Gao, Xi Pei, Xie George Xu

**Affiliations:** ^1^ School of Nuclear Science and Technology University of Science and Technology of China Hefei Anhui China; ^2^ Technology Development Department Anhui Wisdom Technology Company Limited Hefei Anhui China; ^3^ Department of Radiation Oncology The First Affiliated Hospital of the University of Science and Technology of China Hefei Anhui China

**Keywords:** ionization chamber, low‐kV radiotherapy, Monte Carlo simulations, percent depth dose

## Abstract

**Background:**

Kilovoltage x‐ray units (50–150 kVp) are widely used for skin cancer treatments, necessitating precise dosimetric characterization to ensure accurate dose delivery. This study validates the depth dose data of the VF‐80/𝜇XHP (80 kV, 100 W) x‐ray system for use in low‐kV therapy with a contemporary detector.

**Objective:**

This research aims to characterize the kilovoltage VF‐80/𝜇XHP x‐ray unit with open‐ended PMMA and stainless‐steel applicators of various diameters by utilizing different x‐ray energy values.

**Methods:**

Measurements included machine output reproducibility, applicator leakage and thickness effects, measurement of HVL, reference output dosimetry, and applicator‐to‐phantom spacing. PDD curves were obtained using various filter/kV and applicator combinations with a PTW 34013 parallel‐plate ionization chamber in a solid water phantom and through MC simulations with the TOPAS Monte Carlo code in a water phantom, which were validated against literature data.

**Results:**

The study evaluated around 40 x‐ray beams. The PDDs were measured in a solid water phantom using a PTW 34013 ionization chamber and calculated with the TOPAS Monte Carlo code. Both the measured and computed percent depth dose data were compared to the BJR 25 supplementary. The agreement between ionization chamber measurements and MC calculations with BJR 25 supplementary was within [1.9%, 1.5%] for 50 kV x‐rays and within [0.5%, 0.2%] for 70 kV x‐rays.

**Conclusion:**

This research provides the dosimetric analysis of a kilovoltage VF‐80/μXHP x‐ray machine. The primary focus was on using a PTW 34013 ionization chamber to measure the percentage depth dose in a solid water phantom, along with dose calculations performed using the TOPAS Monte Carlo simulation. The findings align well with existing literature, confirming that this x‐ray unit is suitable for low‐kV x‐ray radiotherapy applications.

## 1 INTRODUCTION

Radiation therapy for superficial cancerous or benign lesions has been used for many years, utilizing x‐ray beams with energies ranging from 6 to 300 kilovolts (kV).^1^ The International Atomic Energy Agency (IAEA) recommends clinical use of superficial (50–150 kVp) and orthovoltage (150–500 kVp) radiotherapy.^2^ Despite the availability of electron beams from linear accelerators, kilovoltage (kV) treatment machines are still used in radiotherapy departments, and there is renewed interest in improving equipment design and dosimetric parameters of the x‐ray units.^3^ In many cases, kV radiotherapy offers a simple yet highly effective alternative to megavoltage photon and electron therapies. Besides radiotherapy, kV x‐ray beams are widely employed for diagnostic purposes, including radiography, fluoroscopy, and tomography, as well as in various research applications.^4^, ^5^, ^6^, ^7^ The overlap between diagnostic and therapeutic uses is exemplified by the use of onboard kV imaging on linear accelerators for patient positioning and target localization. Additionally, kV x‐rays are extensively used in preclinical and radiobiology research.^8^, ^9^, ^10^, ^11^ A key feature of superficial kV beams is that the dose peaks at the skin surface and diminishes rapidly with depth due to attenuation and scattering. Consequently, therapeutic kV beams are primarily used to treat skin cancers and manage various dermatological conditions, including keloid scars, mycosis fungoides, and psoriasis. The doses used in superficial radiotherapy can be relatively high and may follow standard or hypo‐fractionated regimens.^12^ However, superficial radiotherapy treatments can cause undesirable acute effects, including skin erythema, depigmentation, and hair loss, making it challenging to balance reducing these toxicities with achieving therapeutic goals. Therefore, accurate dose assessment is essential for successful treatment. One of the primary requirements for precise treatment planning is obtaining parameters such as percent depth dose (PDD) tables for different filter/kV settings and applicator combinations, along with associated dose rates. These parameters are vital for determining the optimal prescription depth in superficial radiotherapy. Despite the apparent simplicity of kV treatments, determining reference and relative doses is not a straightforward process.^13^ When evaluating a kilovoltage therapy unit, relative dosimetry data must be collected to ensure accurate patient dose calculations. Measuring kilovoltage PDDs is challenging due to steep dose gradients and variation in beam energy with depth in low‐energy photon beams. As a result, measuring kilovoltage PDDs requires a chamber with a flat energy response and high spatial resolution.^14^, ^15^, ^16^, ^17^, ^18^ The choice of phantom is critical, as many materials are not tissue‐equivalent at kilovoltage energies. It is recommended to perform measurements in a water phantom using a well‐characterized parallel‐plate chamber to ensure suitability for kilovoltage depth‐dose measurements.^18^ However, practical difficulties arise when measuring dose at the surface with a parallel‐plate chamber in a water phantom. Direct surface dose measurements are difficult because the chamber's front window or waterproof cap extends above the water level, making measurements at depths less than the window or cap thickness impossible. A safety margin is also necessary to prevent collisions between the chamber and the applicator. This margin accounts for the positioning of the applicator at the water surface, ensuring adequate space between the applicator and the ionization chamber.

In this study, we selected the Varex VF‐80/μXHP x‐ray unit for depth dose calculations because no existing research has characterized its depth dose or explored its potential in superficial therapy. Although the VF‐80/μXHP x‐ray tube by Varex Imaging is designed for fluorescent applications, many users employ low‐kV and analytical x‐ray tubes for radiotherapy and research purposes.^19^, ^20^, ^21^ These studies collectively highlight the growing interest and feasibility of using compact low‐kV x‐ray systems like the VF‐80/μXHP in preclinical radiotherapy research. For the dose distribution, we designed applicators made of PMMA and steel. These applicators (5 cm in length, 3 mm wall thickness) were fabricated in‐house and attached directly to the tube's exit window, providing field aperture diameters of 2, 3, 4, or 5 cm at the phantom surface. We quantitatively examined how different applicator materials and thicknesses influence doses from the VF‐80/μXHP x‐ray tube. Depth dose measurements using an ionization chamber and Monte Carlo simulations for the VF‐80/μXHP tube are compared with existing low‐kV x‐ray depth dose literature data.

This study aimed to determine the PDD using open‐ended cone PMMA and steel applicators with the VF‐80/𝜇 XHP x‐ray tube. During the experiment, depth‐dose measurements were performed with a PTW 34013 thin‐window ionization chamber in a water‐equivalent solid water phantom. These measurements were supported by Monte Carlo simulations calculated using the TOPAS Monte Carlo code, employing the x‐ray setup of the VF‐80/𝜇 XHP x‐ray tube to understand dose characteristics at shallow depths. All measurements were compared with the published BJR 25 supplement.1 Additional tests included machine output reproducibility, half‐value layer, back‐scattering factors, applicator leakage, and calibration of the beam output.

## 2 MATERIALS AND METHODS

### 2.1 VF‐80 / μXHP (80 kV, 100 W) and open‐ended applicator

**FIGURE 1:**
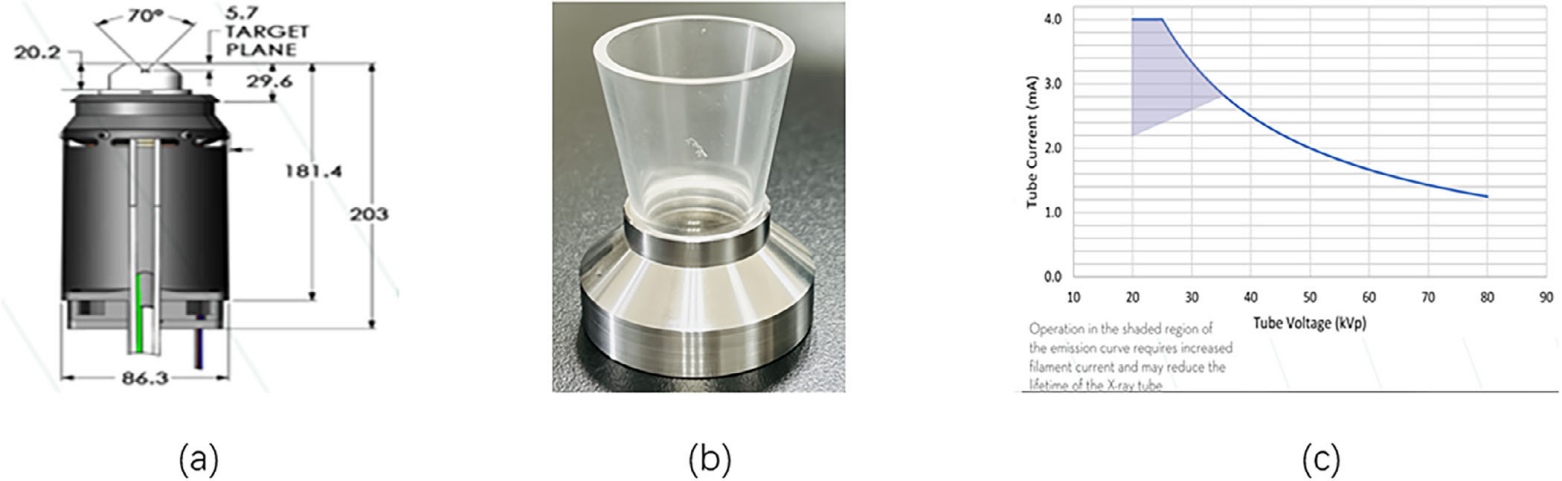
Schematic diagram of (a) VF‐80/uXHP x‐ray tube, (b) PMMA applicator, (c) Vendor's current emission graph.

The VF‐80/μXHP x‐ray tube was made by VAREX Imaging. It features an x‐ray generator that can produce up to 100 W of power and x‐ray energies from 10 to 80 kV, with tube currents from 0.0 to 4.0 mA. In this study, we used a current of 1.0 mA for all x‐ray energies in depth dose calculations. The tube uses a transmission tungsten (W) as the anode target material, angled at 90° and has a focal spot size of 1.0 mm. It has an inherent filtration of 75 µm of beryllium (Be). We customized the VF‐80/μXHP tube with open‐ended cone applicators made of polymethyl methacrylate (PMMA) and stainless steel to focus the beam for therapy‐like applications. These applicators, measuring 5 cm in length with 5 mm wall thickness, were made in‐house and attached directly to the tube's exit window, creating field aperture diameters of 2, 3, 4, or 5 cm at the phantom surface. For this study, we used x‐ray energies of 50 kV/Al‐1.0 mm and 70 kV/Al‐2.0 mm. During PDD measurements for all x‐ray energies, the applicator openings were positioned at the solid water phantom surface to replicate the standard clinical setup, where the applicator is flush against the patient's skin. The VF‐80/μXHP tube, along with the applicator and x‐ray tube current graph, is shown in Figure 1.

### 2.2 Timer reproducibility

The timer reproducibility tests were conducted using in‐air measurements with an applicator of 4 cm in diameter and a PTW 34013 ionization chamber positioned 5.57 cm from the focal spot at the opening of the applicator to air. Reproducibility (*R*) was assessed through five consecutive 1‐min measurements at tube potentials of 50 and 70 kV using Equation 1.

(1) $$R=\frac{\sigma }{M}\times 100\%$$

where *σ* represents the standard deviation, and *M* is the mean charge collected over five measurements. The tolerance for reproducibility (*R*) was set to be ± 0.15%. The reproducibility test was repeated by stopping and restarting the beam during 1‐min exposures at 50 and 70 kV. The effect of interruptions was quantified using Equation 2.

(2) $$R_{i}=\frac{\left(\mathrm{Mint}-M\right)}{M}\times 100\%$$

where *R*_i_ is the reproducibility with interruptions, *M*_int_ is the mean charge collected during five consecutive 1‐min measurements, each with one interruption, and *M* is the mean charge collected from five uninterrupted 1‐min measurements.

### 2.3 Back‐scattering factor

The measurement of the backscatter factor (BSF) in superficial radiation therapy involves quantifying the dose enhancement caused by scattered photon radiation returning to the surface of a medium, such as a water phantom or human tissue. The BSF is defined as the ratio of the dose measured at the surface of a full‐scatter phantom (e.g., water or PMMA) to the dose measured in the same geometry without the phantom (i.e., in air) at the same measurement point.^15^ We utilized a PTW 34013 ionization chamber and the TOPAS Monte Carlo code to evaluate backscatter for 50 and 70 kV x‐rays in both air and a solid water phantom, employing PMMA applicators of varying field diameters (2 , 3 , and 5 cm). To simulate realistic clinical conditions as in superficial therapy, lead shielding was also incorporated adjacent to the field edges. BSFs (Bw) were calculated as a function of source‐to‐surface distance (SSD), field size, HVL (Al mm), and the presence of clinical lead shielding.

(3) $$\mathrm{BSF}=\frac{\mathrm{Dsurface},\;\mathrm{phantom}}{\mathrm{Dsame}\;\mathrm{point},\;\mathrm{in}\;\mathrm{air}}$$

### 2.4 Applicator thickness and applicator‐phantom spacing

The measurements were performed to examine the effect of applicator thickness on the x‐ray spectrum as it passes through the applicator surface and how varying thicknesses of applicators influence the output x‐ray fluence. To investigate the impact of applicator thickness on x‐ray fluence, we used PMMA applicator thicknesses of 3 , 5 , and 10 mm. We also assessed the effect on surface dose of the spacing between the applicator and the solid water phantom surface. The dose measurements were taken by changing the distance between the applicator and the solid water phantom surfaces to determine how much the dose varied with a change in distance. All measurements were performed using a PTW 34013 ionization chamber.

### 2.5 Leakage dose measurements

The PTW 34013 ionization chamber was used to measure leakage dose around the x‐ray unit exit mount and the surface of the PMMA applicator. The setup involved an x‐ray source emitting photons at energies of 50 and 70 kV through a PMMA applicator with diameters ranging from 2 to 5 cm. To measure the leakage dose along the x‐ray tube and applicator, the PTW 34013 ionization chamber was positioned around the applicator and x‐ray unit at various radial and longitudinal distances along the applicator and tube at different points.

### 2.6 Half‐value layer

Central‐axis HVL measurements were performed using a narrow‐beam geometry, following the AAPM TG‐61 protocol.^15^ The setup incorporated a PTW 34013 ionization chamber, designed to minimize primary beam scatter, with the ionization chamber positioned at 100 cm FSD. An aluminum (Al) filter of varying thickness was inserted between the tube and the ionization chamber, as specified in AAPM TG‐61. The measured half‐value layers were compared with the nominal values and the SpekPy software.^22^

### 2.7 Output dose calibration measurements

The absorbed dose to water at the surface was determined using the in‐air method outlined in the AAPM TG‐61 protocol.^15^ A PTW UNIDOSE electrometer and a PTW 34013 ionization chamber were used. The calibration point was set at the applicator‐specific focus‐to‐surface distance (FSD). The absorbed dose to water at the solid water phantom surface,

(4) $$\begin{array}{c} D_{w,x=0}=\left(MP_{\mathrm{TP}}P_{\mathrm{ion}}P_{\mathrm{pol}}P_{\mathrm{elec}}\right)\times N_{k}\times B_{w}\\ \times P_{\mathrm{stem},\mathrm{air}}\times \left[\left(\frac{\mu en}{\rho }\right)\right]w,air \end{array}$$

where *D*_w,x = 0_ is the dose to water at the solid water phantom. The P_TP_P_ion_P_pol_P_elec_ are standard correction factors. *B*_w_ is the BSF, and the quantity in brackets is the ratio of the mass energy‐absorption coefficient. The chamber stem correction factor was assumed to be unity. Temperature and pressure were measured using traceable digital instruments.

### 2.8 Measurements of PDD in the solid water phantom

**FIGURE 2:**
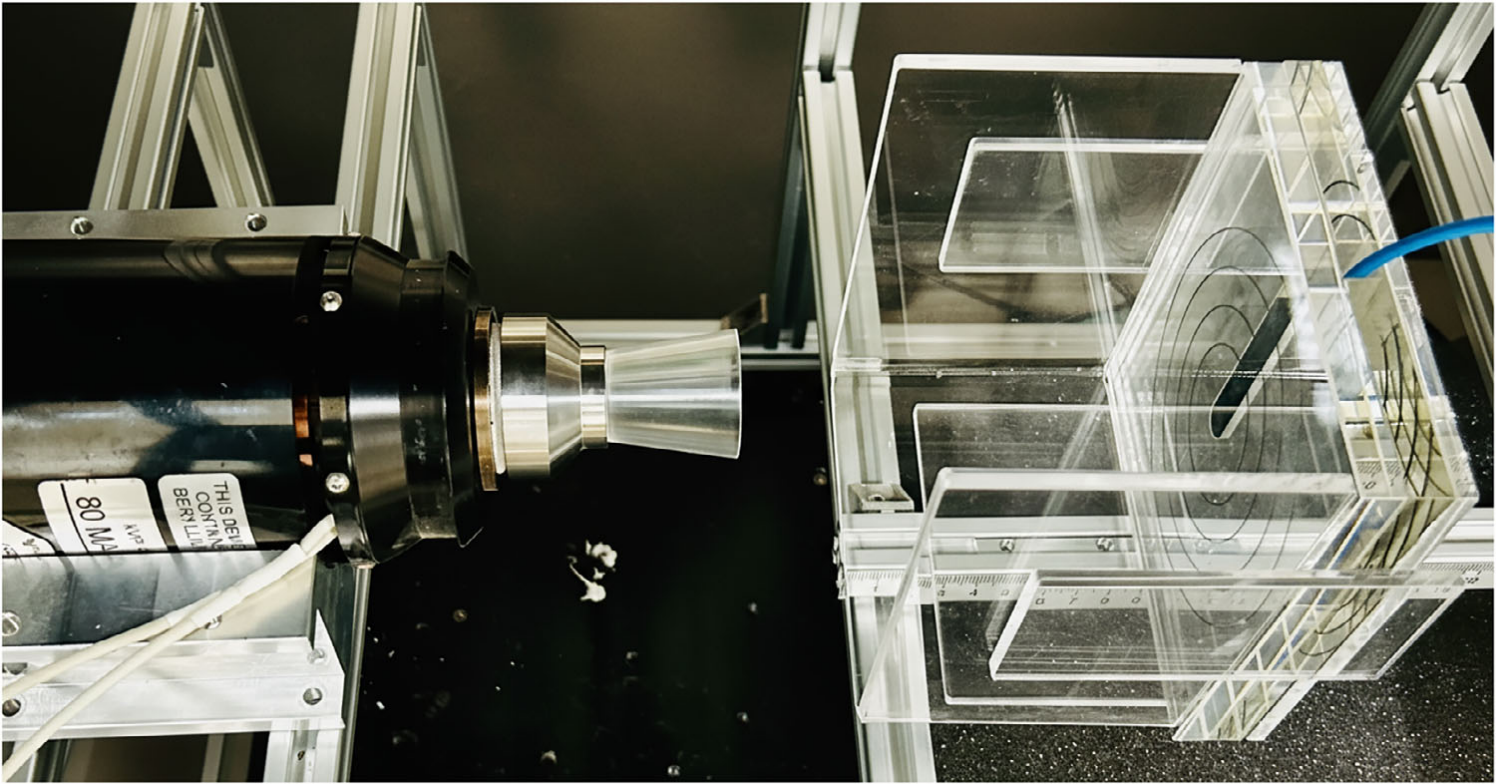
Schematic diagram of measurement setup (x‐ray tube with PMMA applicator, and solid water phantom holding an ionization chamber).

Measurements of PDD were performed using a PTW 34013 ionization chamber in a solid water phantom. According to the vendor's specifications, this chamber has a nominal sensitive volume of 0.005 cm^3^, a window thickness (incident foil thickness) of 0.03 mm of PE, a sensitive volume measuring 1.45 mm in radius with a depth of 0.9 mm, and an electrode radius of 1.4 mm. This chamber applies for low‐energy photons in the energy range of 15–70 kV. The chamber was positioned within a solid water phantom designed to be tissue equivalent for photons in the energy range of 30–70 kV. This material is recommended by Sheu et al as suitable for relative dosimetry measurements in kilovoltage beams.^18^ PMMA applicators, each with a thickness of 3 mm, a length of 5 cm, and diameters ranging from 2 to 5 cm with 1 cm increments, were utilized for dose measurement in a solid water phantom, with a source‐to‐focal spot distance to applicator exit of 5.57 cm. Dose measurements were conducted at depths from the surface of the phantom down to 40 mm, using a 50 mm backscatter material. All measurements were normalized to 100% at a depth of 0.03 mm in the phantom; this value corresponded to the thickness of the incident foil in the chamber. The measurement setup, x‐ray tube with applicator and an ionization chamber in a solid water phantom, is shown in Figure 2.

### 2.9 Monte carlo calculations

Monte Carlo is a numerical method based on statistics, and in radiotherapy, dose calculations using this method are considered the gold standard. Monte Carlo simulations were performed with TOPAS V3.9, an extension of the Geant4 toolkit.^23^, ^24^ TOPAS is an application built on Geant4 that simplifies parameter setting and adjustment. Photon spectra for the VF‐80/μXHP x‐ray tube were created with SpekPy V2.0.8 using tube parameters such as a tungsten target, applied potential (50–70 kV), 75 µm beryllium inherent filtration, and additional filtration of 1.0 mm Al for 50 kV and 2.0 mm Al for 70 kV.^22^, ^25^ The tube has a 1.0 mm focal spot, and the transmission tungsten anode target minimizes the heel effect, producing a nearly symmetric photon distribution even for the largest 5 cm diameter of applicator. The photon emission angle was set at 70°, covering a wide (70°) × long (70°) area. Radiation transport within the target was explicitly modeled using Geant4 low‐energy physics lists (g4em‐Livermore, g4em‐Penelope, and g4em‐Standard‐opt4), including elastic scattering, inelastic collisions, bremsstrahlung, and characteristic x‐ray generation. Cross‐section databases used were EPDL 97 for Livermore, PENELOPE for Penelope, and standard Geant4 data for Standard EM. The transport range cut was 0.000005 µm for all particles, with electrons tracked down to 100 eV. The applicator's distal end was positioned 5.57 cm from the x‐ray focal spot, based on a tube exit‐to‐focal spot distance of 5.7 mm and an applicator length of 5 cm. A water phantom measuring (4 cm depth × 6 cm × 6 cm) was subdivided into voxels of (0.01 mm × 0.1 mm × 0.1 mm), with a source‐to‐surface distance of 5.57 cm. Simulations utilized 10⁹ particle histories and were executed on a server equipped with AMD EPYC 7763 × 2 CPUs, 1024 GB of RAM, and 256 threads, ensuring statistical uncertainties of less than 1%. A statistical uncertainty of less than 1% was achieved in the central‐axis dose voxels used for depth‐dose calculations. All results presented in the manuscript fall within this < 1% statistical uncertainty.

## 3 RESULTS

### 3.1 Timer reproducibility

For the time reproducibility, the 1‐min measurement interval was chosen solely for the reproducibility test and does not reflect typical treatment times, as the machine can operate continuously for hour with stable output. Short intervals, such as 1 min, are commonly used because they allow for multiple consecutive measurements to be taken efficiently while providing statistically meaningful data. The measured timer reproducibility, *R*, as defined by Equation (1), was found to be 0.13% for 50 kV and 0.04% for 70 kV. The reproducibility with intentional interruptions, *R*_i_, as described by Equation (2), was only 0.15% for 50 kV and 0.07% for 70 kV, indicating that disruption during clinical treatment would have a negligible dosimetric effect.

### 3.2 Back‐scattering factor

The literature indicates that a significant BSF occurs near the surface of solid‐water phantoms, resulting in an increased superficial dose. The BSF is defined as the ratio between the dose measured at the phantom's surface (with full scatter conditions) and the dose measured at the same point in free air. This increase is primarily due to photons scattering from surrounding materials back into the measurement region. In this study, we measured BSF values and compared them to those reported in the AAPM TG‐61 report (shown in Table 1). The findings align with expected physical principles: larger applicator diameters produce higher BSFs because a larger area of surrounding material contributes to photon backscatter toward the central axis. Conversely, smaller fields lose a larger portion of scattered photons to the surrounding space, resulting in a lower contribution to the central dose. To closely represent clinical conditions used in superficial therapy, we performed additional measurements and simulations with lead shielding placed next to the radiation field. This setup mimics the clinical use of shielding to protect adjacent healthy tissues. We observed that the introduction of lead shielding, positioned close to the surface and at the edge of the radiation field, slightly altered the BSF values. Specifically, there was a minor reduction in the BSF due to the absorption of low‐energy scattered photons by the lead.

**TABLE 1:** Backscatter factor B_w_ values comparison between this study and the AAPM TG‐61 report.

SSD (cm)	Field diameter *d* (cm)	50 kV/Al1.0 mm	BSF with 2.5 mm of Pb	TG‐61 HVL 1.0 mm	70 kV/Al2.0 mm	BSF with 2.5 mm of Pb	TG‐61 HVL 2.0 mm
5	2	1.096	1.079	1.081	1.117	1.089	1.104
	3	1.112	1.093	1.098	1.145	1.117	1.132
	5	1.121	1.105	1.116	1.179	1.159	1.163

### 3.3 Applicator thickness and applicator phantom spacing

#### 3.3.1 Applicator thickness

We observed that the thickness of PMMA applicators significantly affected the x‐ray dose output at energies of 50 and 70 kV. Dose outputs were measured for applicators with varying diameters, with wall thicknesses of 3 , 5 , and 10 mm. Our results indicate that as the applicator wall thickness increases, the dose output correspondingly decreases. This reduction is due to greater photon beam attenuation within the thicker applicator. For smaller‐diameter applicators, the reduction in dose output was notably more pronounced. This effect occurs because the applicator walls are positioned closer to the beam axis, leading to increased photon attenuation at the edges and greater scattering effects. An additional observation was the higher PDD at the surface when using smaller‐diameter applicators. This phenomenon may arise not only from increased photon scatter but also from electron contamination, as noted in Section VII C of the AAPM TG‐61 report. Specifically, electrons generated from interactions within the applicator's inner walls can more readily enter the central beam axis when the walls are positioned closely, as is the case with small‐diameter applicators. Such electron contamination artificially elevates the surface dose, contributing distinctly from photon backscatter alone. To mitigate and differentiate this electron contribution from photon components, TG‐61 (Section III B) about thin surface absorbers can be used. These absorbers can effectively eliminate contaminating electrons, ensuring that surface dose measurements more accurately represent the photon component. We evaluated dose distributions with 3 , 5 , and 1 cm thick PMMA endplates to quantify the optimal thickness. The data suggest that a 5 mm thickness provides minimal secondary electron contribution, and at the same time, a minimal electron absorber effectively stops radiation leakage while maintaining good dose output. This thickness balances electron suppression with efficient dose delivery. Table 2 illustrates the differences in percentage dose outputs for applicators with 3 mm wall thickness compared to those with 5  and 10 mm thicknesses. As an example, for a 50 kV x‐ray beam and a 2 cm diameter applicator, increasing the wall thickness from 3  to 5 mm resulted in a 14% reduction in dose output, while increasing to 10 mm thickness resulted in a significant 34% dose reduction.

**TABLE 2:** Percentage dose difference (%) with applicator thickness of 3–5 , 3–10 , and 5–10 mm.

		Energy	
Applicator diameter	Applicator thicknesses	50 kV	70 kV
2 cm	3 mm/5 mm	14 %	9 %
	3 mm/10 mm	34 %	24 %
	5 mm/10 mm	24 %	17 %
3 cm	3 mm/5 mm	12 %	8 %
	3 mm/10 mm	29 %	21 %
	5 mm/10 mm	19 %	14 %
4 cm	3 mm/5 mm	9 %	7 %
	3 mm/10 mm	19 %	15 %
	5 mm/10 mm	11 %	9 %
5 cm	3 mm/5 mm	5 %	4 %
	3 mm/10 mm	8 %	7 %
	5 mm/10 mm	4 %	3 %

#### 3.3.2 Applicator‐phantom spacing

During clinical radiotherapy procedures, the applicator surface is ideally placed in full contact with the patient's skin. However, any separation between the applicator and the skin surface alters the delivered dose due to geometric divergence [following the inverse square law (ISL)] and a potential reduction in contaminant electron contribution. To evaluate this effect, we measured dose output at various air gaps between the applicator and a solid water phantom using a 5 mm thickness PMMA applicator. When a 1 mm gap was introduced, we observed dose reductions of approximately 3% for 2 and 3 cm diameter applicators, 2.4% for the 4 cm applicator, and 1.8% for the 5 cm applicator. At a 10 mm separation, the dose dropped by 26% for the 2 cm applicator, 24% for 3 cm, 21% for 4 cm, and 14% for 5 cm diameter. These results are consistent with ISL predictions, but may also be influenced by the loss of low‐energy electrons that contribute to surface dose during contact measurements. As described in Section VII C of AAPM TG‐61, these contaminant electrons, generated from the applicator material or air, can significantly affect surface dose. Their contribution diminishes rapidly with even small air gaps, which may partly explain the steep drop in measured dose near the surface. To distinguish between the photon dose and electron contamination, the use of thin surface absorbers recommended in TG‐61 Section III B may be incorporated in measurements. These absorbers would help isolate the true photon component by filtering out secondary electrons, thereby improving the accuracy of surface dose evaluation.

### 3.4 Leakage dose measurements

The leakage dose from the x‐ray unit head with the applicator was calculated using a PTW 34013 ionization chamber. The leakage dose around the PMMA applicator of varying diameters was measured at various radial distances from the outer wall surface of the applicator, and the results were analyzed as a function of the central axis dose of the applicator center. Given that the thickness of the applicator and the x‐ray distribution were homogeneous along its length, we calculated the leakage at different points around the applicator. When the leakage of radiation was measured longitudinally, the leakage dose was highest at the interface between the x‐ray unit and the applicator, decreasing as we moved toward the exit opening of the applicator. Around the surface of the applicator at different radial distances, the highest dose was recorded at the applicator's surface and diminished with increasing radial distance from that surface. The radial dose distribution exhibited a rapid decline with distance, consistent with the expected attenuation in the water phantom. During the leakage radiation calculations, the background radiations were also measured and subtracted from the applicator‐measured leakage dose. To improve the accuracy of results, the measurements were repeated five times. At a radial distance of 0.5 cm from the applicator surface, the leakage dose was only about 0.65% of the central dose for an x‐ray energy of 50 kV and approximately 1.03% for 70 kV. However, at the point of the applicator that attached to the x‐ray tube, the longitudinal leakage dose was about 2.4% compared to the central dose of the applicator for 50 kV and 3.8% for 70 kV x‐ray energy and at the applicator middle point this leakage was only 0.34% for 50 kV and 0.65% for 70 kV compared to central dose of the applicator. The over all uncertainty in the measurements was about 1.8%.

### 3.5 Half‐value layer

The measured half‐value layers following the AAPM TG‐61 are compared with the nominal and SpekPy‐calculated half‐value layers, represented in Table 3. For the used filters, the SpekPy Half‐value layers were within 0.01 mm relative to the measured half‐value layer values. The agreement between all measured and nominal half‐value layers was within 4%. The observed half‐value layer differences are acceptable.

**TABLE 3:** Summary of the beam voltage, tube current, and quality parameters in use.

Tube voltage (kVp)	Tube current (mA)	Added filtration	Nominal HVL	SpekPy HVL	Measured HVL
50	1	1.0 mm Al	1.0 mm Al	0.95 mm Al	0.94 mm Al
70	1	2.0 mm Al	2.0 mm Al	2.0 mm Al	2.04 mm Al

### 3.6 Output dose calibration

The ratio of mass energy‐absorption coefficients was obtained from AAPM TG‐61, using the recommended values for the measured HVLs (1.020 for 50 kV, HVL = 1.0 mm Al; 1.018 for 70 kV, HVL = 2.0 mm Al). Additionally, spectrum‐averaged values were calculated by weighting NIST data with the generated x‐ray tube spectra. The in‐air calibration adhered to AAPM TG‐61 protocols, with an estimated standard uncertainty of ± 2.1% for 50 kV and ± 2.7% for 70 kV. Thus, TG‐61 values were used in the dose determination for consistency with protocol recommendations, and the independent spectral averaging confirmed their accuracy in the context of our study. Four applicators and two x‐ray energy beams at 50 and 70 kV created eight beam combinations, with dose rates listed in Table 4. As expected, the measured dose rates at a given focal source distance increased gradually with larger applicator sizes due to increased backscatter.

**TABLE 4:** Beam dose rate in air under reference conditions on VF‐80/μXHP.

		Dose in air (Gy/min)	
Applicator diameter (cm)	Focal source distance FSD (cm)	50 kV	70 kV
2	5.57	2.137	2.376
3	5.57	2.159	2.401
4	5.57	2.165	2.409
5	5.57	2.183	2.431

### 3.7 Beam data

Depth dose distributions for x‐ray energies 50 and 70 kV with different diameters of PMMA/stainless steel applicators are described in the following sections.

#### 3.7.1 Dose distribution with PMMA applicators

**FIGURE 3:**
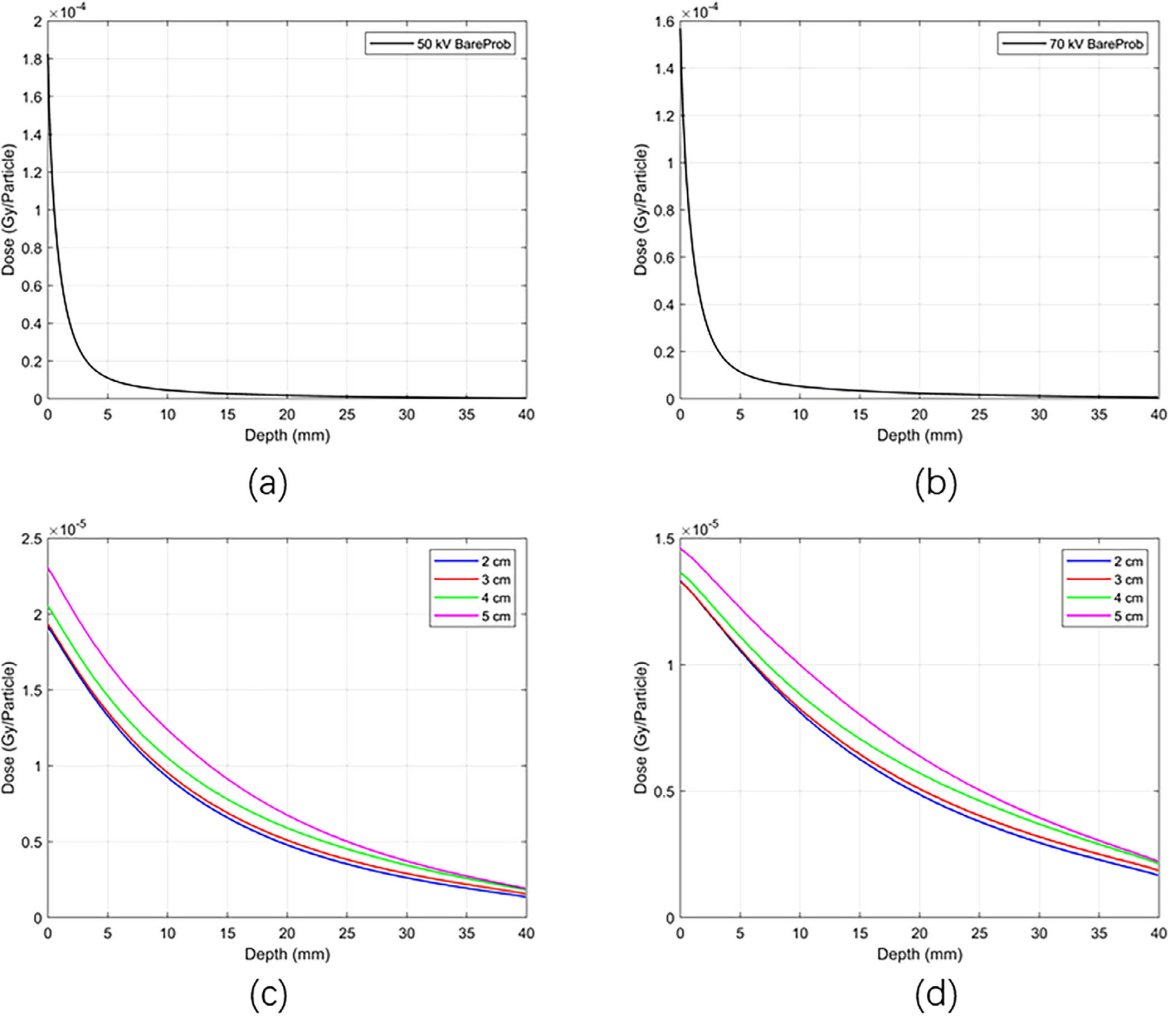
Absolute depth dose distributions of the x‐ray beams of (a) 50 kV bare prob, (b) 70 kV bare prob, (c) 50 kV with PMMA applicators, and (d) 70 kV with PMMA applicators using MC simulations.

**FIGURE 4:**
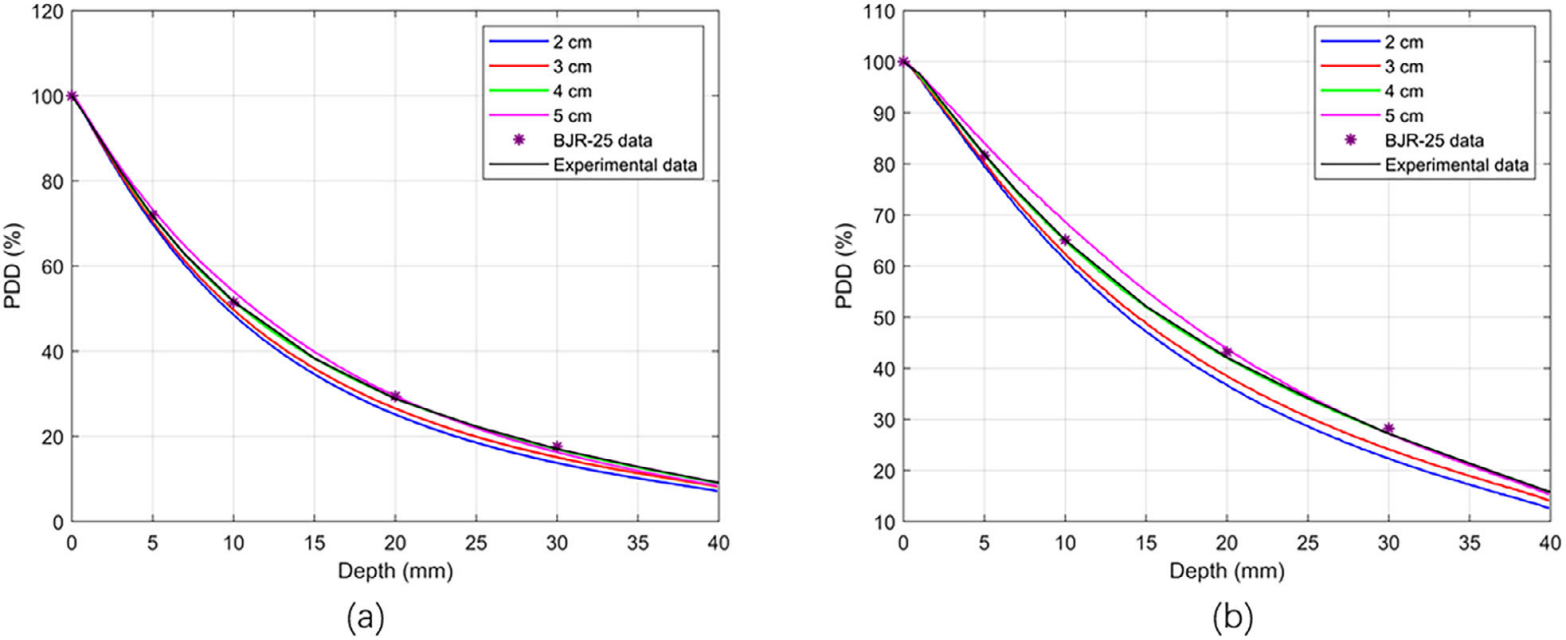
Comparison between measured (experimentally with IC), calculated (TOPAS MC), and BJR 25 supplementary percentage depth dose distribution with PMMA applicators (a) 50 kV, (b) 70 kV x‐ray energies.

Figure 3a–d displays the absolute depth dose distributions for 50 and 70 kV x‐ray energies using MC simulations, respectively, using bare probes and applicators with diameters of 2 , 3 , 4 , and 5 cm, each with a thickness of 5 mm. It is clear that as the energy increases from 50 to 70 kV, the surface dose decreases for each applicator diameter. Figure 4a,b shows the PDD distributions for the 50 and 70 kV x‐ray beams, using applicators with diameters ranging from 2 to 5 cm. The PDD measurements were performed using both TOPAS MC and a PTW 34013 ionization chamber. The color‐coded lines represent dose distributions simulated by the TOPAS MC code, while the black line corresponds to experimental PDD data measured with a 4 cm diameter applicator using an ionization chamber in a solid water phantom. Violet stars indicate depth dose data sourced from the literature, specifically the BJR 25 supplement. PDD trends varied with applicator size: smaller applicators showed higher percentages near the surface, whereas larger applicators had higher values at deeper depths. The MC‐calculated PDD closely matched the experimental measurements. Both the MC simulation and experimental data align well with the literature's BJR 25 supplement. For 50 kV energy (Figure 4a), the difference between experimental and simulated data was approximately 2%, while the deviation between experimental results and the BJR 25 supplement was about 1.5%, and the difference between Monte Carlo calculations and the BJR 25 supplement was around 1.9%. At 70 kV (Figure 4b), the divergence between experimental and simulated dose distributions was approximately 1.31%, the divergence between experimental data and the BJR 25 supplement was 0.2%, and the difference between Monte Carlo calculations and the BJR 25 supplement was roughly 0.5%.

#### 3.7.2 Isodose distribution of PMMA applicators

**FIGURE 5:**
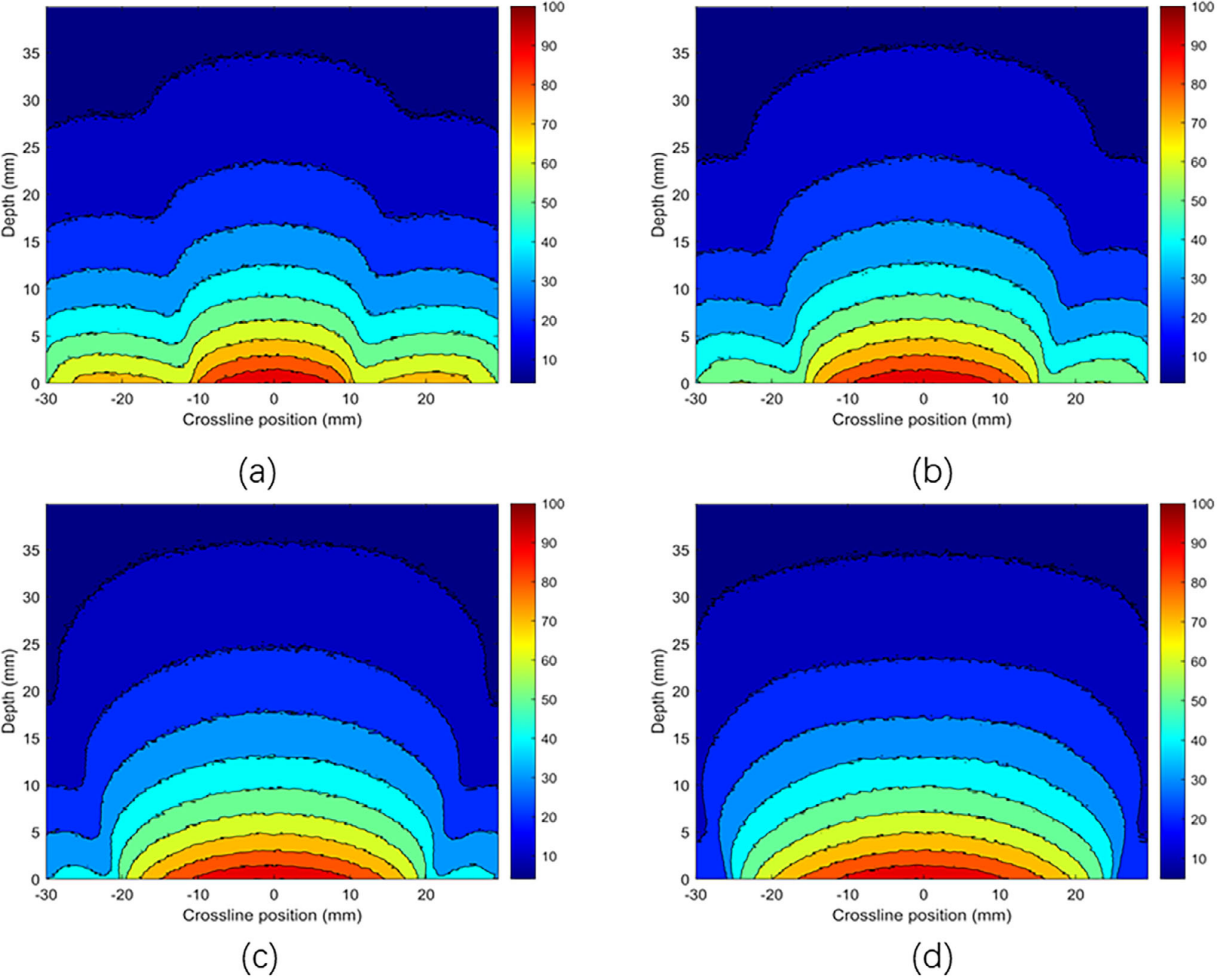
Dose distribution of 50 kV x‐ray beam with PMMA applicators using MC simulations with (a) 2 cm diameter, (b) 3 cm diameter, (c) 4 cm diameter, and (d) 5 cm diameter of applicators.

**FIGURE 6:**
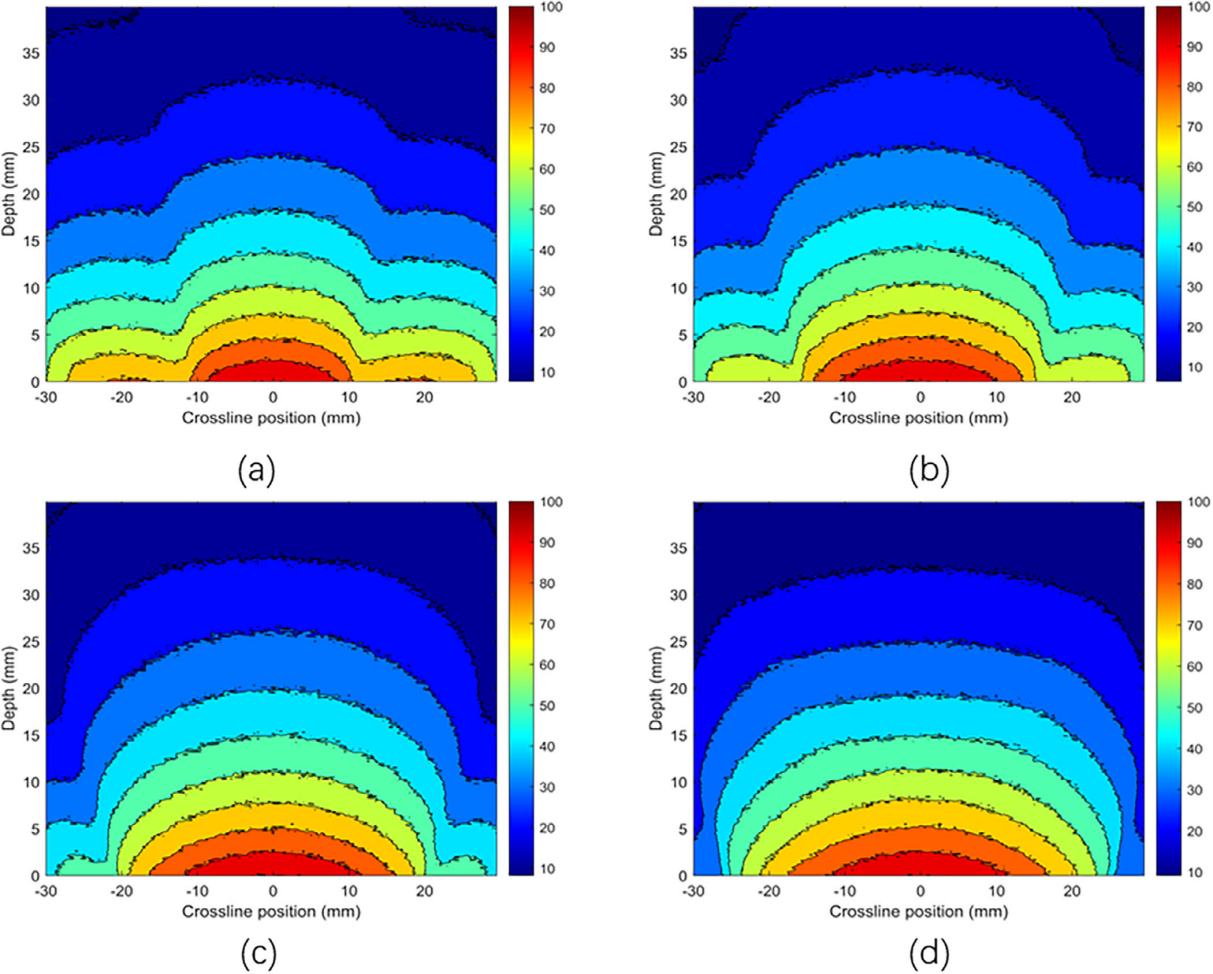
Dose distribution of 70 kV x‐ray beam with PMMA applicators using MC simulations with (a) 2 cm diameter, (b) 3 cm diameter, (c) 4 cm diameter, and (d) 5 cm diameter of applicators.

The dose distributions for various photon energies were carefully evaluated at different depths and along a crossline within a water phantom using MC simulations. The phantom was irradiated using applicators with diameters from 2.0  to 5.0 cm and x‐ray energies of 50 and 70 kV, as illustrated in Figures 5 and 6. For the 70 kV (Figure 6) x‐ray beam with a 5.0 cm diameter applicator, the dose was ≥90% along the cross‐line profile extending ± 13 mm laterally and approximately 3 mm longitudinally along the depth. In contrast, with a 4.0 cm diameter applicator, the dose remained ≥90% within ± 12 mm across the cross‐line profile and approximately 3 mm along the depth direction. For the 2.0  and 3.0 cm diameter applicators, the dose sustained levels of ≥90% within ± 8  and ± 11 mm along the cross‐line profile, respectively, and approximately 2.7  and 2.3 mm along the depth direction. It is important to note that the penumbra, referring to the dose ratio at a point off the central axis to the dose on the central axis, increases with increasing depth. This phenomenon can be attributed to the absorption and divergence of the photon beams as they traverse through the water phantom, leading to decreased dose uniformity with depth.

#### 3.7.3 Dose distribution with stainless‐steel applicators

**FIGURE 7:**
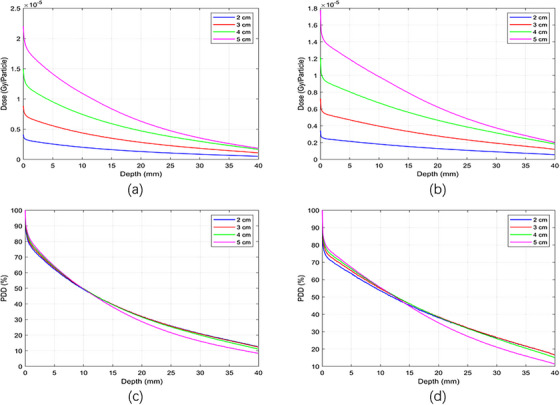
Absolute depth dose using x‐ray beam of energies (a) 50 kV and (b) 70 kV, and the percent depth dose (PDD) with x‐ray beams of (c) 50 kV and (d) 70 kV with different diameters of stainless‐steel applicators using MC simulations.

In this study, we initially calculated the dose distributions of x‐ray beams using PMMA applicators placed within a solid water phantom, employing an ionization chamber and simulations with the TOPAS Monte Carlo code. We now present an analysis of the x‐ray dose distribution using stainless steel applicators, modeled solely by using Monte Carlo simulations. Monte Carlo simulations with stainless steel applicators were performed exclusively for comparison with PMMA applicators to evaluate the effect of applicator material on percentage depth dose distributions. These results are presented for methodological comparison only and are not intended for clinical application. These results are purely MC simulations with open‐ended steel applicators with a steel endplate. Figure 7a,b shows the absolute depth dose distributions for 50 and 70 kV x‐ray energies, respectively, across stainless steel applicators with diameters of 2 , 3 , 4 , and 5 cm, each 5 cm long and 3 mm thick. It was observed that as the x‐ray energy increased, the absolute surface dose decreased for each applicator diameter. Additionally, larger‐diameter applicators showed a relatively higher dose compared to smaller ones. The dose distribution at the water phantom surface with stainless steel applicators differed from that of PMMA applicators, with the absolute dose being lower across all diameters for the stainless‐steel applicators. Specifically, for a 50 kV (Figure 7a) x‐ray using a 5 cm steel applicator, the surface dose was approximately 2.5% lower than that with a PMMA applicator of the same size. As the steel applicator diameter decreased, the dose differences became more pronounced. However, as the x‐ray energy increased, the dose difference between steel and PMMA applicators decreased. Figure 7c,d depict the percentage relative dose distribution for 50 and 70 kV x‐ray energies using TOPAS MC, respectively, with stainless steel applicators of different diameters. The relative dose with the smaller‐diameter applicator exceeds that of all larger diameters from 0  to 1 mm depth across all beam energies. From 1  to 3 mm depth, the dose from the 5 cm diameter applicator was higher. Beyond 3 mm in depth, the dose delivered by the 2 and 3 cm diameter steel applicators surpasses that of the other sizes.

#### 3.7.4 Isodose distribution of steel applicators

**FIGURE 8:**
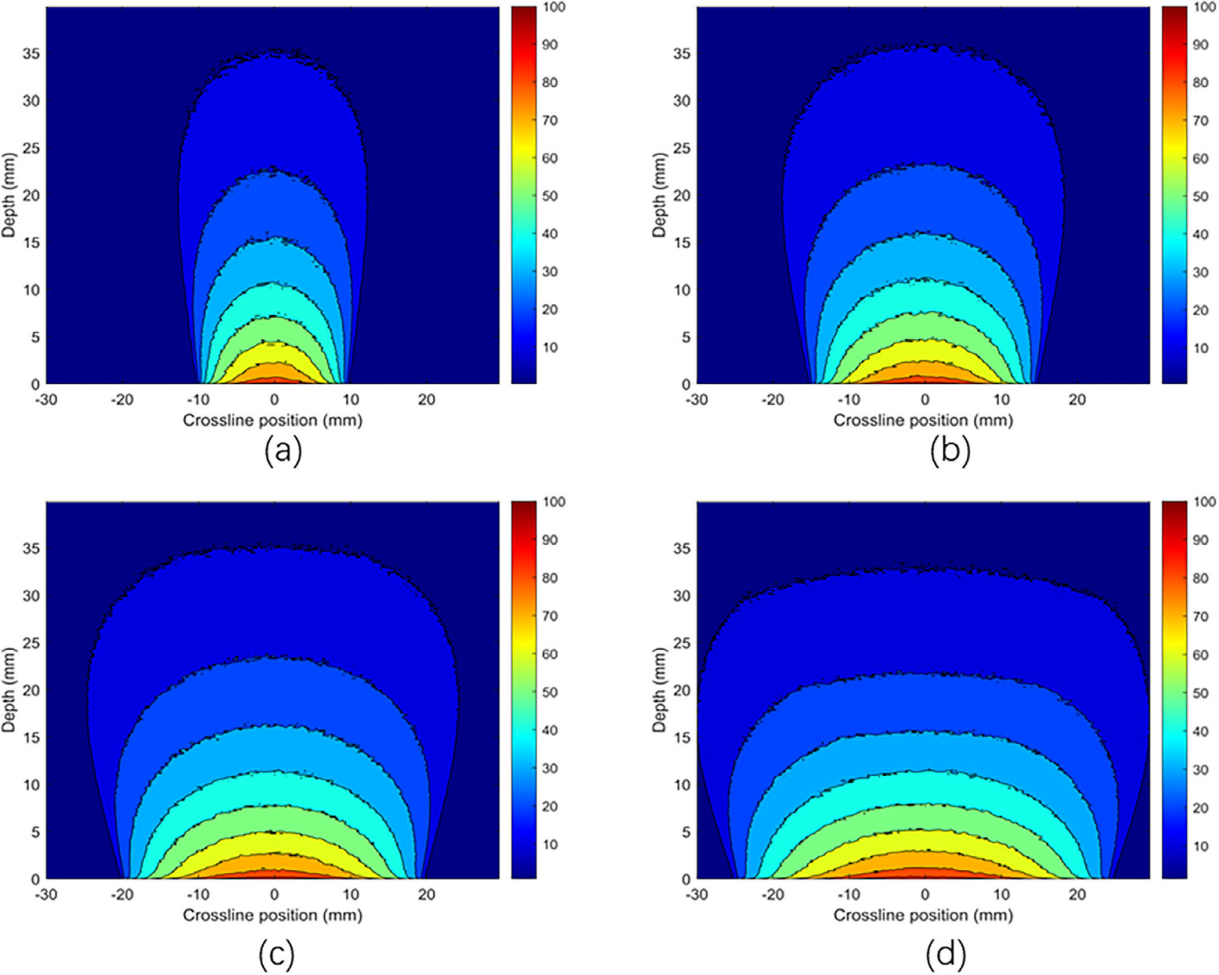
Dose distribution with MC simulations of a 50 kV x‐ray beam using stainless steel applicators of (a) 2 cm diameter, (b) 3 cm diameter, (c) 4 cm diameter, and (d) 5 cm diameter applicators.

**FIGURE 9:**
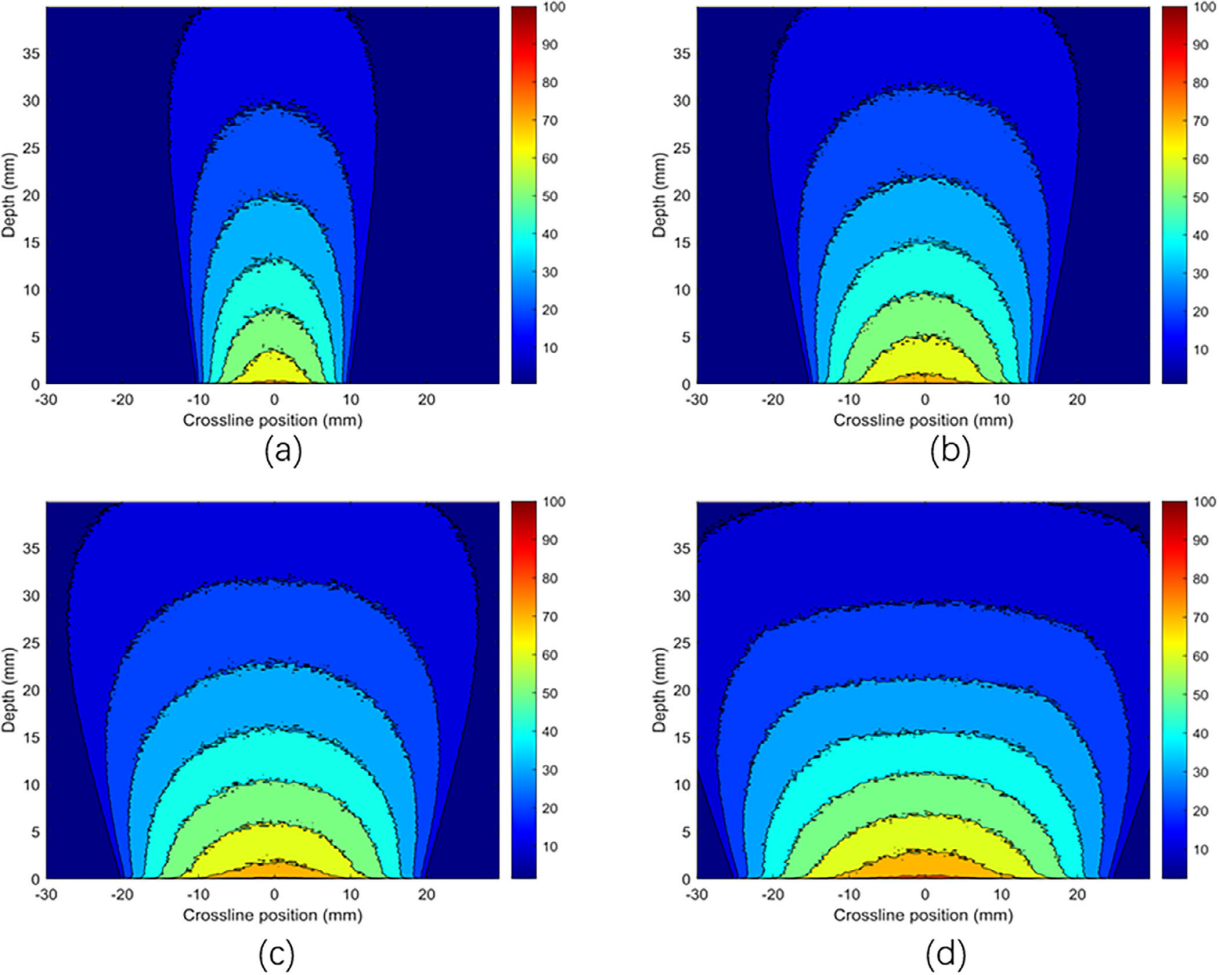
Dose distribution with MC simulations of a 70 kV x‐ray beam with stainless steel applicators of (a) 2 cm diameter, (b) 3 cm diameter, (c) 4 cm diameter, and (d) 5 cm diameter applicators.

Figures 8 and 9 illustrate the dose distribution of x‐ray beams with energies of 50 and 70 kV at various depths and along a crossline within a water phantom, utilizing steel applicators with diameters of 2.0 , 3.0 , 4.0 , and 5.0 cm using MC simulations. The 50 kV (Figure 8) x‐ray beam achieves a relative dose distribution with a minimum of 80% for a 5.0 cm diameter applicator, maintaining this threshold within a ± 11 mm range laterally and approximately 1.3 mm in depth. For the 4.0 cm diameter applicator, the relative dose distribution stays at or above 80% within a ± 9 mm off‐axis range and about 1 mm in depth. Similarly, for the 2.0  and 3.0 cm diameter steel applicators, the relative dose distribution remains at or above 80% within ± 4  and ± 7 mm off‐axis, respectively, and 0.3  and 0.5 mm in the depth axis. These measurements underscore the consistency of dose distribution across different applicator sizes despite variations in diameter. A decrease in dose at the surface occurs as energy increases, as indicated by the x‐ray beams' enhanced penetration at higher energies, resulting in a reduced surface dose. The figures visually represent this phenomenon, providing valuable insights into the behavior of x‐ray beams at varying energies and their interaction with the phantom. These findings are essential for optimizing radiotherapy treatment plans, ensuring effective dose delivery to the target area while minimizing exposure to surrounding tissues.

## 4 DISCUSSION

The purpose of this study was to present PDD data from the VF‐80/μXHP x‐ray tube. Efforts were made to acquire PDD data at x‐ray energies of 50 and 70 kV in a solid water phantom using a PTW 34013 ionization chamber experimentally and with TOPAS Monte Carlo calculations in a water phantom, employing four different applicator diameters (made of PMMA and stainless steel). This work quantitatively analyzed the impact of applicator material and thickness on therapeutic dose, and also evaluated the leakage dose around the machine and applicator. The reduction in surface dose with increasing energy was attributed to the reduced backscatter and increased penetration of higher‐energy x‐rays. The differences observed between PMMA and stainless‐steel applicators were mainly due to the higher attenuation coefficient of steel. Specifically, for stainless‐steel applicators, a significant dose spike was observed at the water phantom surface, which was less pronounced with PMMA applicators (Figure 7c,d). This difference was due to higher contamination electron contribution from steel applicators at the surface, along with the sharp dose drop caused by radiation absorption within a few millimeters at the surface of the phantom. PMMA applicators, in contrast, produced smoother distributions without such pronounced effects (Figure 4). Applicator size also influenced dose: smaller applicators showed higher surface doses due to contaminant electrons, while larger applicators demonstrated higher doses at depth due to increased backscatter. For x‐ray energies in the kV range, liquid water is the preferred medium for reference dosimetry and PDD measurements.^15^ The differences in PDD compared to liquid water include ‐21.7% for Plastic Water and +17.6% for polystyrene at 50 kVp. Generally, the water equivalence of solid water phantoms depends on energy, with dosimetric variations decreasing as the kV beam energy increases. Most commercially available solid water phantoms are water‐equivalent within ± 2% in the kV range. In this study, we used a solid water phantom that was equivalent to water.^3^ For absolute and percentage depth dose measurements at low‐energy x‐rays, thin‐window, small‐volume plane‐parallel ionization chambers are commonly used.^15^ In this study, a PTW 34013 parallel plate ionization chamber was employed for PDD measurements in a solid water phantom. Ionization chambers are not ideal for dose measurements near the water surface; the minimum measurement depth should be at least the chamber's outer radius to avoid surface perturbations and detector response differences between air and water.^3^ Using a PTW 34013 ionization chamber, dose measurements were performed at 0.03 mm, which is the thickness of the chamber's front window. The PDD was measured for different energy values and quality settings of the VF‐80/μXHP x‐ray unit, as reported by Sheu et al., since beam quality can vary between units.^18^ Consequently, PDD will differ for each x‐ray unit. Dose measurements in a solid water phantom with a PTW 34013 ionization chamber showed good agreement with TOPAS MC calculations, with average relative dose differences of 2% at 50 kV and 1.31% at 70 kV. The experimental PDD data collected in a solid water phantom and the MC‐calculated PDD in a water phantom were comparable to data from the literature, BJR‐25 supplementary. The average relative dose difference between experimental and Monte Carlo data from BJR‐25 was [1.9%, 1.5%] for 50 kV and [0.5%, 0.2%] for 70 kV. Our findings are consistent with previously published Monte Carlo and experimental studies. In particular, Verhaegen et al. presented PDD data for kilovoltage x‐ray beams (50–300 kVp) in water.^26^ Their results demonstrated steep dose fall‐off with depth, with PDD values around 45%–50% at 10 mm and 15%–20% at 30 mm for low kVp beams like 60 kVp, closely matching our measured and simulated data using PMMA applicators. At higher energies, they reported reduced surface dose and deeper penetration, which was also observed in our 70 kV results. The quantitative comparison shows that our results are ∼2.3% smaller for 50 kV compared to Verhaegen et al. 60 kV energy and ∼1.53% higher for 70 kV compared to Verhaegen et al. 60 kV energy. The results confirm that applicator material and size significantly influence dose distribution and highlight the importance of considering these factors in low‐energy radiotherapy treatment planning.

### 4.1 PDD measurements at depths less than 0.1 mm

**FIGURE 10:**
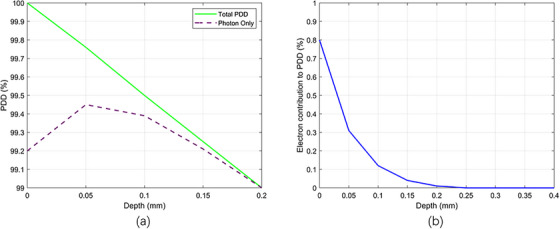
(a) Comparison of PDD curves with and without contaminant electrons for a 2 cm diameter PMMA applicator at 50 kV. (b) Contribution of contaminant electrons to the percentage depth dose (PDD) as a function of depth in water for a 2 cm diameter PMMA applicator at 50 kV.

A significant contributor to surface dose in kilovoltage x‐ray beams is electron contamination originating from the treatment applicators and the machine head. These secondary electrons increase dose deposition at very shallow depths. In this study, the smallest applicator exhibited the most pronounced contamination effect, consistent with its geometry and closer proximity to the central axis, which enhances electron fluence toward the surface. The AAPM TG‐61 protocol provides specific recommendations on ionization chamber wall thickness and buildup materials to minimize such effects.^15^ The PTW 34013 parallel‐plate chamber used in our measurements has a 0.03 mm polyethylene entrance window; an additional 0.1 mm BoPET (Mylar) buildup material is recommended to adequately suppress electron contamination. Evidence of contamination became clear in our full Monte Carlo simulations of the tube and applicator. In these simulations, the surface dose was defined as the dose to the center of the shallowest voxel (0.01 mm). When simulations were performed using only the generated x‐ray spectrum (photon only), electron contamination from the unit and applicator was not included. Under these conditions, a sharp reduction in dose was observed between the surface (0 mm) and 0.1 mm depth, creating an apparent “build‐up” shape (Figure 10a, dashed line), as noted by Nahum and Knight.^27^ However, this effect is not a true photon build‐up phenomenon as seen at megavoltage energies, but rather an artifact caused by neglecting electron contamination. When contaminant electrons were included, the near‐surface dose increased (Figure 10a, solid line), in agreement with experimental expectation. Figure 10b further quantifies the electron contribution, showing that their effect is largest at the surface (∼0.8% of total PDD) and diminishes rapidly, becoming negligible beyond 0.2 mm. This demonstrates that electron contamination is the primary cause of the elevated surface dose, and omission of these electrons can lead to underestimation of photon dose at clinically relevant depths.

### 4.2 Limitations and future work

This study examined dose distribution only using open‐ended applicators. Closed‐end applicators, which may be used for kilovoltage radiotherapy and are effective at preventing scattered radiation exposure outside the target area, were not included. Dose measurements were performed with water‐equivalent materials (solid phantoms) and did not involve actual patient tissues like skin or breast tissue, since heterogeneities in tissue cause different dose distributions. Although solid water phantoms provide a reasonable approximation, differences compared to liquid water were observed, indicating that the phantom material can influence the absolute dose. Future work will include measurements in liquid water to more accurately verify dose distributions and further validate Monte Carlo simulations and RBE assessments for the Vf‐80/μXHP x‐ray unit with open‐ended applicators.

## 5 CONCLUSION

This study provides dosimetric data obtained from the VF‐80/μXHP x‐ray unit, emphasizing the assessment of PDD in a solid water phantom. The PDD was measured using a parallel plate PTW 34013 ionization chamber. The research findings demonstrate a strong agreement between the PDD profiles determined through ionization chamber measurements in the solid water phantom and those calculated by TOPAS Monte Carlo simulations. The PDD measurements with the chamber and the calculated PDD with MC align well with the BJR 25 supplementary. The outcomes of this study affirm that the VF‐80/μXHP x‐ray system is suitable for low‐kV therapy applications.

## AUTHOR CONTRIBUTIONS

Muhammad Zeeshan: Writing, review, & editing, writing original draft, validation, methodology, formal analysis, data curation, and conceptualization. Yikai Wu: methodology, formal analysis. Zhongyu Qi: review and experimental dose calculations. Ning Gao: Review & editing. Pie Xi: Formal analysis. Xie George Xu: Writing—review & editing, formal analysis, conceptualization.

## CONFLICT OF INTEREST STATEMENT

The authors reported no potential conflict of interest.

## ETHICS STATEMENT

This study did not involve human participants or animals. All simulations and the experimental data used in this study were obtained in compliance with institutional safety and ethical standards. No ethical approval was required.

## Data Availability

Data will be made available from the corresponding author on request.
